# Effect of ultrasound-guided injection of botulinum toxin type A into shoulder joint cavity on shoulder pain in poststroke patients: study protocol for a randomized controlled trial

**DOI:** 10.1186/s13063-024-08258-8

**Published:** 2024-06-27

**Authors:** Peng Zheng, Yu Shi, Hang Qu, Meng lin Han, Zhi qiang Wang, Qing Zeng, Manxu Zheng, Tao Fan

**Affiliations:** Department of Rehabilitation Medicine, Zhujiang Hospital, Southern Medical University, Guangzhou, Guangdong China

**Keywords:** Stroke, Botulinum toxin type A, Hemiplegic shoulder pain, Randomized controlled trial, Protocol

## Abstract

**Background:**

Hemiplegic shoulder pain (HSP) is a common complication after stroke. It severely affects the recovery of upper limb motor function. Early shoulder pain in hemiplegic patients is mainly neuropathic caused by central nerve injury or neuroplasticity. Commonly used corticosteroid injections in the shoulder joint can reduce shoulder pain; however, the side effects also include soft tissue degeneration or increased tendon fragility, and the long-term effects remain controversial. Botulinum toxin injections are relatively new and are thought to block the transmission of pain receptors in the shoulder joint cavity and inhibit the production of neuropathogenic substances to reduce neurogenic inflammation. Some studies suggest that the shoulder pain of hemiplegia after stroke is caused by changes in the central system related to shoulder joint pain, and persistent pain may induce the reorganization of the cortical sensory center or motor center. However, there is no conclusive evidence as to whether or not the amelioration of pain by botulinum toxin affects brain function. In previous studies of botulinum toxin versus glucocorticoids (triamcinolone acetonide injection) in the treatment of shoulder pain, there is a lack of observation of differences in changes in brain function. As the content of previous assessments of pain improvement was predominantly subjective, objective quantitative assessment indicators were lacking. Functional near-infrared imaging (fNIRS) can remedy this problem.

**Methods:**

This study protocol is designed for a double-blind, randomized controlled clinical trial of patients with post-stroke HSP without biceps longus tenosynovitis or acromion bursitis. Seventy-eight patients will be randomly assigned to either the botulinum toxin type A or glucocorticoid group. At baseline, patients in each group will receive shoulder cavity injections of either botulinum toxin or glucocorticoids and will be followed for 1 and 4 weeks. The primary outcome is change in shoulder pain on the visual analog scale (VAS). The secondary outcome is the assessment of changes in oxyhemoglobin levels in the corresponding brain regions by fNIRS imaging, shoulder flexion, external rotation range of motion, upper extremity Fugl-Meyer, and modified Ashworth score.

**Discussion:**

Ultrasound-guided botulinum toxin type A shoulder joint cavity injections may provide evidence of pain improvement in patients with HSP. The results of this trial are also help to analyze the correlation between changes in shoulder pain and changes in cerebral hemodynamics and shoulder joint motor function.

**Trial registration:**

Chinese clinical Trial Registry, ChiCTR2300070132. Registered 03 April 2023, https://www.chictr.org.cn/showproj.html?proj=193722.

## Background and rationale {6a}

Hemiplegic shoulder pain (HSP) is a relatively common complication after stroke, with a prevalence of 16–84% [1]. HSP usually occurs within 12 weeks after stroke, which can seriously affect the recovery of upper limb motor function [2] and reduce the quality of life of patients [3]. Previous studies have shown that the pathogenesis of HSP is considered to be multi-factor, and paralysis is considered as the main cause of shoulder pain [4]. The current academic opinion suggests that HSP is more likely to be caused by neuropathic pain, which is caused by dysfunctional transmission and signal processing of the nervous system [5, 6].

At present, the treatment methods for HSP include local massage, physiotherapy, motor function training, and oral drugs [7, 8], among which local injection of glucocorticoids is considered a good way to reduce shoulder pain [9], and ultrasound-guided injection increases safety and efficacy [10]. However, the side effects of glucocorticoids injection also include soft tissue degeneration or increased tendon fragility [11]. Botulinum toxin local injection is a new way in HSP treatment [12]. Botulinum toxin acts directly on sensory neurons, which can modulate both muscle hyperactivity and pain caused by sensory impairment due to neurological damage. As a new treatment for pain, Botox does not cause side effects such as degeneration of the tendons and ligaments around the shoulder joint. Related studies have confirmed that injection of botulinum toxin into the muscles surrounding the shoulder joint (especially the subscapularis or pectoralis major) can reduce hemiplegic shoulder pain symptoms by decreasing muscle tone [13–15]. However, intramuscular injection of botulinum toxin has limitations such as exacerbating muscle atrophy [16], dose degradation [17], and unsuitable for patients with low muscle tension [18]. On the other hand, intra-articular drug injections have shown its simplicity, efficacy, safety, and ease of acceptance by patients [19, 20]. The mechanism of joint injection is mainly thought to block the conduction of pain receptors [21] in the shoulder joint cavity, reduce neurogenic inflammation by inhibiting neuropathic pain-inducing substances, and prevent peripheral and central sensitizing factors [22]. However, intra-articular botulinum toxin injections for HSP was rarely seen so far. In previous studies of botulinum toxin versus glucocorticoids in the treatment of shoulder pain, there is a lack of research on the mechanisms of the two drugs in improving pain and a lack of studies observing changes in brain function and the degree of muscle atrophy associated with treatment.

Visual analogue scale (VAS) is the main method used to evaluate pain. However, due to the lack of quantitative indicators as a basis for objective evaluation of HSP, some patients have poor cooperation with subjective evaluation. On the other hand, the existing assessment cannot analyze the mechanism of treatment. Therefore, we choose functional near-infrared imaging (fNIRS) as a secondary evaluation indicator. fNIRS is a technique for non-invasive measurement of cortical hemodynamics. fNIRS systems can monitor changes in local oxyhemoglobin concentration (HBO) caused by neurovascular coupling mechanisms [23]. To assist us in understanding how the brain works, we analyzed the areas of the brain that are activated during specific tasks. A correlation between cortical activation status and pain in patients with osteoarthritis of the knee has been found by fNIRS [24]. In another study of knee osteoarthritis using fNIRS, an increase in cortical blood flow activity was found to be elicited with increased knee pain [25]. Theoretical studies suggest that shoulder pain with stroke patients can be attributed to alterations in the central system associated with persistent pain in the shoulder joint and that persistent pain may induce reorganization of cortical sensory or motor centers [26]. The prefrontal cortex is thought to be the main brain region involved in pain modulation and pain activation [27, 28], receiving bottom-up pain input [29]. In Becerra et al., they used fNIRS to observe hemodynamic changes in bilateral prefrontal and sensory cortex during painful stimuli in healthy subjects [30]. Yucel et al. concluded that the increase in HbO in bilateral primary sensory area (S1) brain regions was only associated with injurious painful stimuli and not with non-injurious stimuli [31]. Related studies have confirmed that by fNIRS signal analysis, pain-related activation regions can be identified in the prefrontal cortex due to the fact that pain stimuli can induce an increase in both oxyhemoglobin and total hemoglobin content in the prefrontal cortex bilaterally [32]. The mechanism of improvement of hemiplegic shoulder pain by different drugs is not clear, and it has not been studied whether shoulder injection therapy affects the activity of different brain regions.

In our study, the aim is to investigate whether intra-articular injections of botulinum toxin into the shoulder cavity are more effective than glucocorticoids in improving pain. fNIRS assessment can increase our understanding of the mechanism of botulinum toxin treatment for HSP and can better provide an objective and reliable basis for clinical treatment.

### Objectives {7}

The primary aim is as follows: to investigate whether shoulder joint cavity injection of botulinum toxin compared with corticosteroids (tretinoin acetate) significantly improves shoulder pain in patients with HSP without biceps tendinitis or acromionic bursitis.

The secondary aims are as follows: in patients with HSP without tendon synovitis or acromioclavicular bursitis, does shoulder joint cavity injection of botulinum toxin type A significantly affect cerebral hemodynamics, improve Fugl-Meyer upper limb motor function, improve passive shoulder motion, and improve Ashworth scale scores compared to glucocorticoids (tretinoin acetate)?

### Trial design {8}

We are planning to conduct a single-center, randomized, double-blind, active-controlled clinical trial. Eligible participants will be randomly allocated in a 1:1 ratio into either the experimental group or the control group. The experimental group will be injected with botulinum toxin in the shoulder joint cavity, and the control group will be injected with glucocorticoids. The trial protocol will follow the Standard Protocol Item: Recommendations for Interventional Trials (SPIRIT 2013) guidelines.

## Methods and analysis

### Study setting {9}

**Fig. 1:**
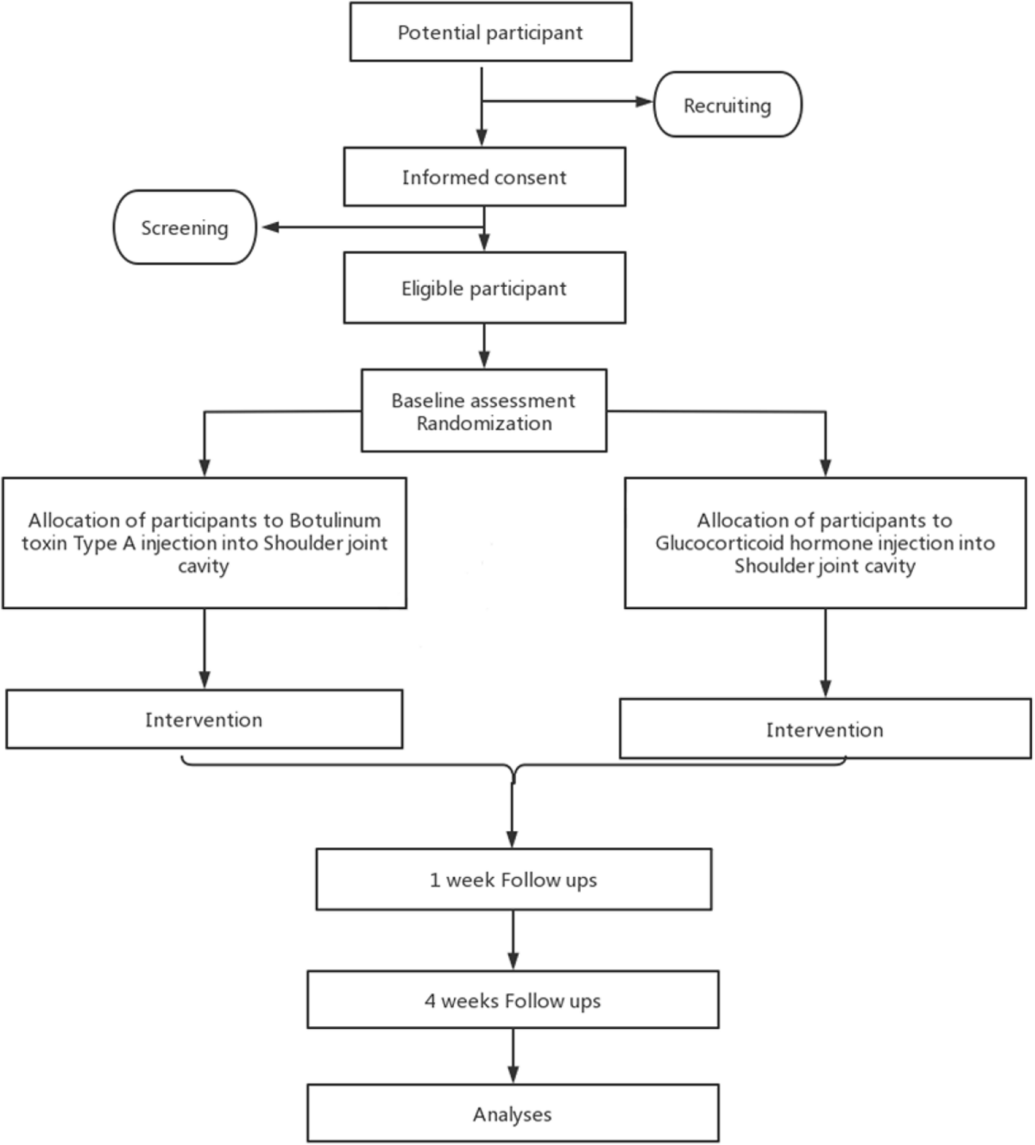
Flowchart of the trial

The study is a 4-week randomized, double-blind controlled trial (Fig. 1). The research center is the Zhujiang Hospital of Southern Medical University, which has received the ethics approval (reference number:2022-KY-269–03). All patients in this study will be required to provide informed consent prior to data collection. Trials will be reported in accordance with CONSORT guidelines.

### Eligibility criteria {10}

Inclusion criteria:

1. Conform to the diagnostic criteria of stroke in Diagnostic Essentials of Major Cerebrovascular Diseases in China 2019; the diagnosis was confirmed by craniocerebral CT or MRI examination.
2. First onset of stroke, age 18–80 years old, duration ≤ 6 months.
3. Visual analog scale (VAS) for pain during passive abduction or external rotation of shoulder joint ≥ 4 points.
4. Shoulder joint drug injection was not performed in half a year.
5. The modified Ashworth scale for external rotation or abduction of shoulder joint was rated between 0 and 1 + 
6. Ultrasound of shoulder joint: the patient showed no accompanying acromion-deltoid slide bursitis or biceps brachialis tendinitis.
7. There are no other bone, joint, and muscle diseases or other neurological diseases that significantly affect motor function.
8. No obvious communication and understanding obstacles, able to carry out normal communication and cooperate with the completion of the experiment.
9. The vital signs are stable and can cooperate with the examination.

Exclusion criteria.

1. llergy to botulinum toxin or glucocorticoid.
2. Intra-articular glucocorticoid or botulinum toxin injection of shoulder in the last 6 months.
3. Previous history of shoulder joint surgery, frozen shoulder, rotator cuff injury, and shoulder joint trauma.
4. Have other diseases more painful than hemiplegia shoulder pain.
5. Active malignant tumors.
6. Coagulopathy, diabetes mellitus, gastric ulcer, infection (within the last 6 months).
7. Currently taking oral glucocorticoids, non-steroidal anti-inflammatory drugs, or immunosuppressive drugs.
8. Pregnant and lactating women.
9. With severe cognitive impairment, Minimum Mental State Examination (MMSE) score less than 21.
10. Those who have not signed the informed consent.

### Who will take informed consent? {26a}

Participants are briefed about the trial before the trial began, and if the patient consented, a written informed consent stating that the patient willing to participate will be obtained.

### Additional consent provisions for collection and use of participant data and biological specimens {26b}

Not applicable. The study used primarily subjective scales to assess efficacy, and the trial did not involve collection of biospecimens for storage.

## Intervention

### Explanation for the choice of comparators {6b}

Local injection of botulinum toxin is an effective method for the treatment of shoulder pain. In order to reduce the time and energy spent in the hospital, we selected a single injection of botulinum toxin to verify its value in the treatment of hemiplegic shoulder pain.

### Intervention description {11a}

Patients will be randomly assigned to experimental group (botulinum toxin) or a control group (triamcinolone acetate). Each group received an injection at baseline. The experimental group will be given the injection of botulinum toxin in the shoulder joint cavity, the injection dose is 100 IU, and 2 ml normal saline will be mixed in advance, that is, the total drug is 2 ml. The control group will receive injections of triamcinolone acetate into the shoulder joint cavity, and 2 ml of total drug will also be injected into the shoulder joint cavity of the control group, including 1 ml of triamcinolone acetate and 1 ml of normal saline.

**Fig. 2:**
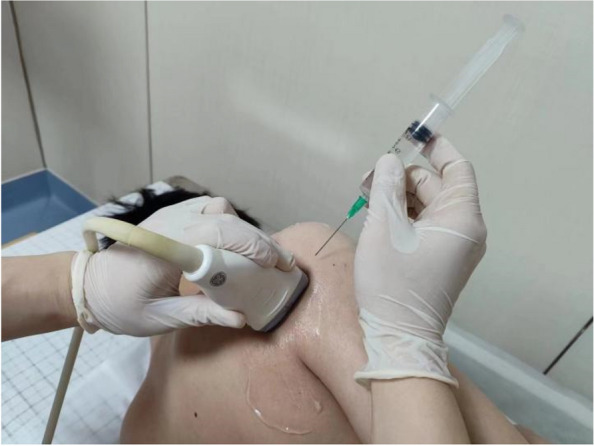
Position diagram of ultrasonic probe and needle

**Fig. 3:**
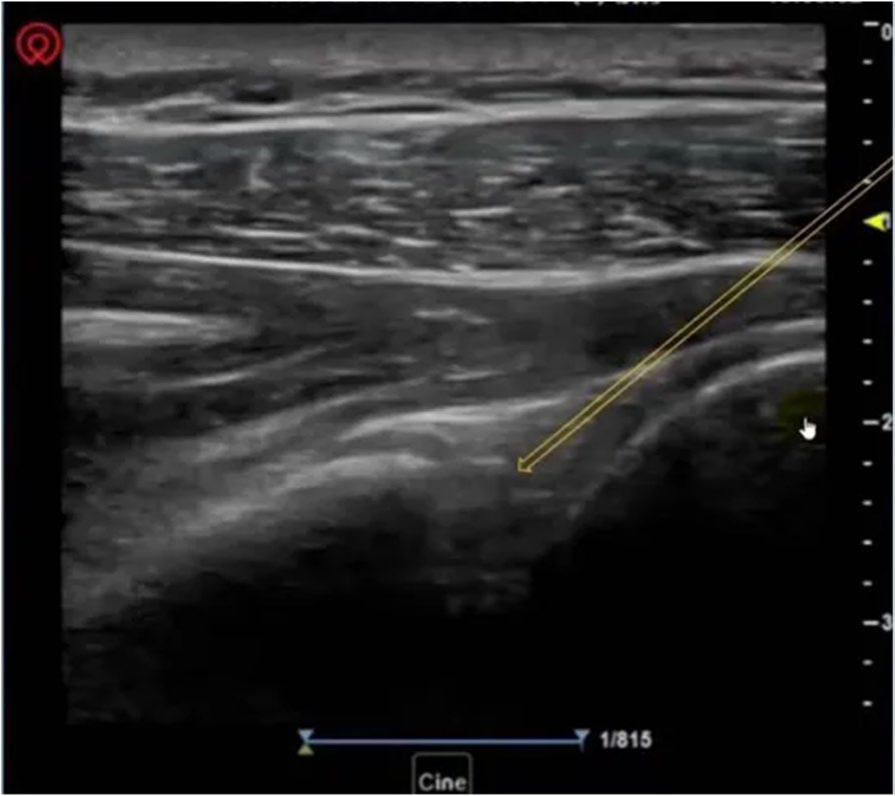
Ultrasound-guided intra-articular injection of shoulder

**Fig. 4:**
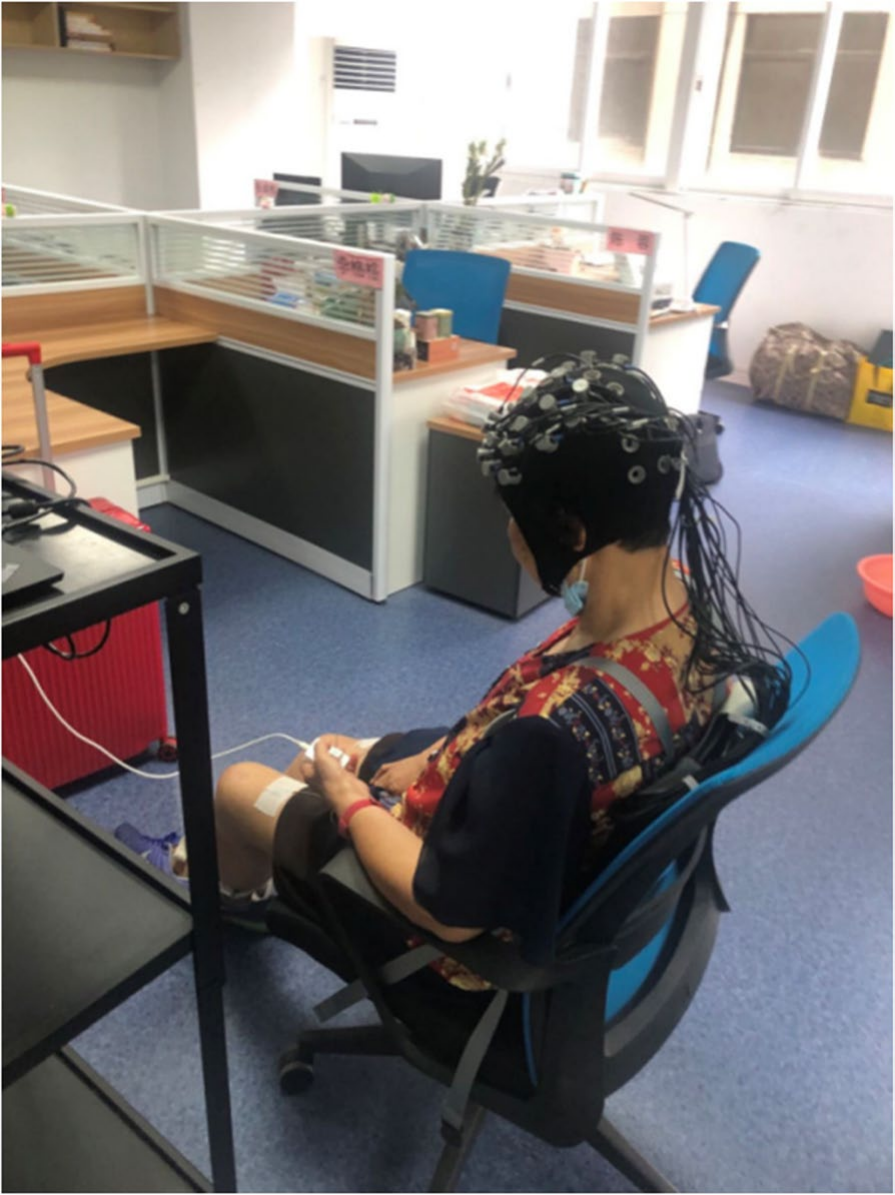
The fNIRS test process

Injections of botulinum toxin or triamcinolone acetate into the shoulder joint cavity will be operated under ultrasound guidance. The process is as follows: (1) patient in lateral position, the affected shoulder to the top, and the affected hand is placed on the healthy side of the chest; (2) the ultrasound probe is placed transversely under the scapular spine and swept anteriorly and posteriorly to reveal the infraspinatus muscle and the articular glenoid; (3) routinely disinfect the towel, hold the probe in one hand, hold the syringe in the other hand, use the lower part of the external scapular spine as the entry point (Fig. 2), and enter the needle inwards (Fig. 3); (4) place the needle in the shoulder joint cavity under ultrasound guidance and slowly push the drug.

### Criteria for discontinuing or modifying allocated interventions {11b}

There will be no special criteria for discontinuing or modifying allocated interventions.

## Strategies to improve adherence to interventions {11c}

In this study, all treatments will be performed by the investigators. All treatments will be recorded and reported in the adherence instructions.

### Relevant concomitant care permitted or prohibited during the trial {11d}

During the trial, injections of any analgesics or oral analgesics into the shoulder are not allowed.

### Provisions for post-trial care {30}

If participants are injured or suffer any discomfort as a result of this study, they are entitled to free treatment and/or compensation for injuries related to this clinical study in accordance with Chinese law.

### Outcomes {12}

All assessments (VAS, upper limb Fugl-Meyer scores, passive range of motion (ROM) improvement, spasticity (MAS) improvement) and information regarding painkiller use will be applied at the baseline and 1-week and 4-week follow-up visits. The VAS pain assessment is the level of pain experienced by the patient during the past week of passive shoulder motion. fNIRS assessments include dorsolateral prefrontal area (DLPFC), primary motor area (M1), primary sensory area (S1), and oxyhemoglobin versus total hemoglobin levels, which will be checked at baseline and 1-week and 4-week follow-up. Other measures including demographics, history taking, and ultrasound of the shoulder joint in the study will be recorded at screening only. Adverse events are documented as described in the results.

#### *VAS* score

Shoulder pain will be assessed using a 100-mm VAS (1 point per 1 mm). Shoulder pain will be measured under passive abduction and passive external rotation. The standard question used is “How would you rate the pain level in your shoulder joint during this movement?”.

#### fNIRS assessments

This experiment used a portable near-infrared functional brain imager (NirSmart, Danyang Huichuang Medical Equipment Co., Ltd., China) to record the cortical activation state. Our equipment consisted of 13 light sources and 15 detectors, making up a total of 35 channels, with an average distance between light sources and detectors of 2.7 cm. Reference was made to the international 10–20 system for localization [33], with the Cz point and Fpz point marked as standard localization points.

Subjects carry a special backpack to carry the fNIRS mainframe which transmits the data to the computer via wireless mode. Based on previous studies, the present study selected bilateral DLPFC, M1, and S1, which have been shown to be associated with sensory function, for testing. In this study, we examined bilateral PFC and S1 brain regions associated with sensory functions, and their channels corresponded to cortical distributions in CH17, CH20, CH22, CH26, CH27, CH31, CH36, CH43, CH48, and CH49. During the fNIRS test, the room temperature is kept at a comfortable constant, and the room is kept quiet. The patient is guided by audio to remain quietly in a seated position (Fig. 4) and is informed that self-activity and communication are prohibited during the test. Measurements will be taken in the resting state and in the shoulder joint external rotation and abduction state, three times in each state and averaged.

Data analysis for this experiment focused on oxyhemoglobin (HbO) concentration as a marker of cortical activation, as it is the most sensitive and reliable indicator of changes related to regional brain oxygenation movements. The NirSpark software is used to analyze the fNIRS data. Oxygenated hemoglobin concentrations for each region are superimposed and averaged to produce results for that region. By analyzing the trend of each parameter, it is possible to see the variation of each channel in the task state versus the resting state.

#### Upper limb Fugl-Meyer scores

The Fugl-Meyer motor function assessment with the upper limb contained 33 items for assessment. This test only requires the assessment of the patient’s upper limb motor function: (1) presence or absence of reflex activity; (2) flexor synergism; (3) extensor synergism; (4) activity with synergism; (5) activity out of synergism; (6) hyperreflexia; (7) wrist stability; (8) elbow extension with 30° of shoulder flexion; (9) fingers; (10) synergism and speed (finger finger-nose test 5 times in a row) 10 items, each with different sub-items, each with a score of 0–2.

#### Shoulder ROM

1. Passive anterior flexion (0° to 170°)

Position: sitting or supine (humerus in neutral position).

Goniometer placement: the axis is located at the lateral crest of the humerus, the fixed arm is parallel to the trunk, and the movable arm is parallel to the humerus.

2. Passive abduction (0° to 180°)

Position: seated or prone (humerus in externally rotated position).

Goniometer placement: the axis is located posterior to the acromion, the fixed arm is parallel to the trunk, and the movable arm is parallel to the humerus.

3. Passive external rotation (0° to 90°)

Position: sitting or supine (90° elbow flexion, 90° shoulder abduction, forearm rotation posterior).

Protractor placement: axis at the eminence, fixed and mobile arms parallel to the forearm. Note: The fixed arm remains in its original position parallel to the ground when the shoulder is externally rotated, while the mobile arm follows the forearm.

#### Spasticity (MAS)

This is a scale for grading muscle tone and assessing spasticity based on passive joint movement resistance. The speed of movement is the completion of a joint movement in 1 s. There are 5 levels: grade 0, no muscle tone; grade 1, a slight increase in muscle tone, passive flexion, and extension of the affected part with minimal resistance or sudden catch and release at the end of the joint range of movement; grade 1 + , a mild increase in muscle tone with sudden catch in the second 50% of the joint range of movement and then minimal resistance in the second 50% of the joint range of movement; grade 2, a more pronounced increase in muscle tone with a more pronounced; grade 3, severe increase in muscle tone, with difficulty in passive movement; grade 4, tonicity, with stiffness and immobility of the affected part during passive flexion and extension.

### Participant timeline {13}

The participant timeline is presented in Table 1.

**Table 1:**
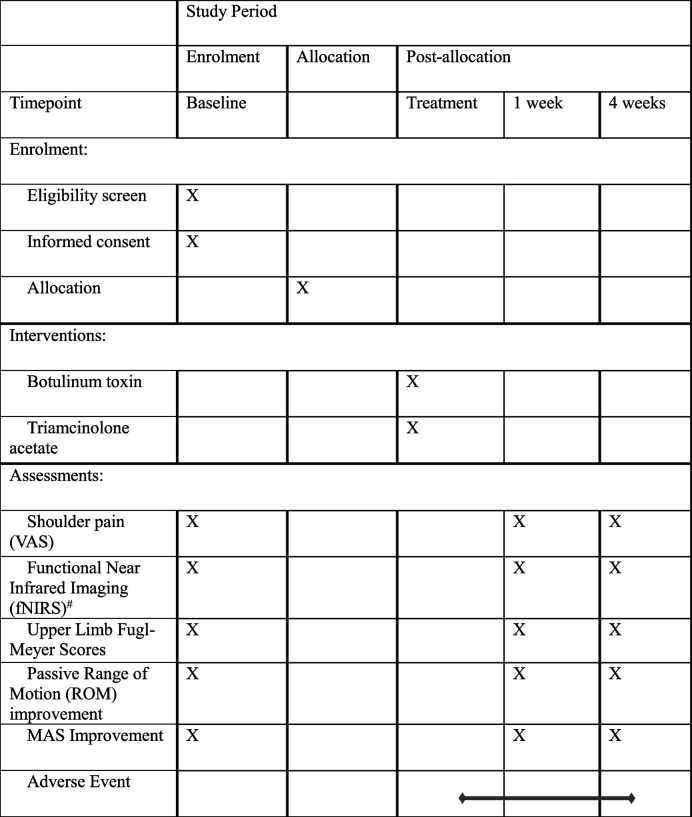
Participant timeline

*VAS*, Visual analogue scale

^#^Functional near-infrared imaging examination includes the primary motor area (M1), dorsolateral prefrontal area (DLPFC), and primary sensory area (S1)

### Sample size {14}

This study is a randomized controlled trial with botulinum toxin injections in the experimental group and glucocorticoid (tretinoin acetate) injections in the control group. The main regression indicator observed is the VAS pain score.

In this study, the sample size will be calculated with reference to similar relevant literature and pre-experimental results. The sample size will be calculated using two independent sample mean validity tests using the G-Power version 3.1.2 software. It is assumed that the means of the two groups are equal at baseline, that there is a 1-point difference in VAS pain score between the two groups at the first post-treatment assessment, and that the standard deviation of the two groups was 1.4, which should be consistent at each subsequent assessment, measured as an effect size (i.e., Cohen *d*, 0.71). Based on these magnitudes, alpha = 0.05 two-tailed is set. To achieve 80% efficacy, 32 patients per group will be counted as study subjects. To compensate for potential loss to follow-up, approximately 20% of patients will be added, with a final inclusion target of 78 patients (39 in each group).

### Recruitment {15}

Participants will be recruited through the following: (1) advocacy by participating clinical teams for presenting patients with hemiplegic shoulder pain, (2) mobilizing the surrounding community hospitals to publicize, (3) posting recruitment advertisements in hospitals and surrounding communities, (4) use social media to post and disseminate e-recruitment advertisements.

## Assignment of interventions: allocation

### Sequence generation {16a}

This study, subjects will be divided into experimental and control groups in a 1:1 ratio using a completely randomized method. Random numbers are generated using the SPSS software, a random allocation table will be prepared, and the drugs are blindly packed according to the random allocation table. The subject number corresponded to the corresponding random number, drug label, and drug name. Everything is to be written on a piece of paper and sealed in an envelope.

### Concealment mechanism {16b}

Allocation concealment will be ensured; the randomization code will not be generated until the patient has completed all baseline measurements. The drugs will be packaged and sealed uniformly by a person unrelated to the trial. Each of the random number assigned are to be written on a piece of paper and enclosed in a sealed envelope.

### Implementation {16c}

Trial assistants will generate and maintain the allocation sequence but will not be involved in the overall treatment process. The principal investigator will group participants according to their number. Participants will not be allowed to change their group assignment after allocation.

## Assignment of interventions: blinding

### Who will be blinded {17a}

This trial is a double-blind design, with subjects and investigators (including outcome measures and statisticians, etc.) not aware of the grouping. The physician who injected the drug is unblinded in this trial as the test drug used in this trial is a colorless, clear liquid with a different appearance to the cloudy glucocorticoid liquid used in the control group. This physician is not involved in the rest of the trial and must be out of the view of the subject and investigator when preparing the drug and injecting it.

### Procedure for unblinding if needed {17b}

There is an emergency unblinding contingency letter, which is numbered to match the subject number and contains instructions for the study drug grouping. If a serious adverse event occurs requiring emergency unblinding, the investigator will open the blinded envelope with the appropriate subject number to determine the drug grouping of the subject.

## Data collection and management

### Plans for assessment and collection of outcomes {18a}

Data collection will be conducted by questionnaire and automatically stored in a mobile database. The database will be collaboratively managed by the research team. Subsequent data analysis will be performed by the statistical analyst, ensuring that both the allocation and the intervention protocol remain blinded.

### Plans to promote participant retention and complete follow-up {18b}

To improve patient retention, we planned a short-term follow-up (4 weeks) to maximize the completeness of data collection and minimize the risk of dropout.

### Data management {19}

#### Data collection

Two testers will assess each subject on each of the scales in strict accordance with the requirements of the test. The “Basic Profile Questionnaire” and the results of each scale for each subject should include the number, date of assessment, and name of each subject.

#### Data recording

The original medical record and the CRF results should be recorded truthfully and carefully as required and the contents should not be changed once completed. If a correction is required due to a genuine error, the original record should not be altered, but only by means of an additional narrative, signed and dated by the responsible study physician.

All results from clinical trials are verified in detail and documented as early as possible to ensure that the data is authentic and reliable.

The various equipment, instruments, and drugs used in clinical research should have strict quality and safety standards and be guaranteed to be used under normal conditions.

### Confidentiality {27}

To ensure privacy, each patient will be assigned an ID number to hold personal information and contact details.

### Plans for collection, laboratory evaluation, and storage of biological specimens for genetic or molecular analysis in this trial/future use {33}

No biological specimens will be collected in the present study.

## Statistical methods

### Statistical methods for primary and secondary outcomes {20a}

All statistical inferences will be performed by two-sided test, the test level of statistical significance is set as 0.05, and the confidence interval of parameters is 95%. The count data describe the number of cases and percentage, and the measurement data describe the mean, standard deviation, minimum, maximum, median, and 25th and 75th quantiles. The counting data give the frequency distribution and the corresponding percentage. Qualitative data give the number of cases of positive rate, positive number, and denominator.

Shapiro–Wilk test will be used to verify the normality of the variables. For normally distributed variables, continuous variables are represented by “mean ± standard deviation”; for data that is not normally distributed, it is represented by “median, interquartile.”

In the analysis of baseline data, the independent sample *t* test will be used to compare the normal distribution between the measurement data groups. The non-normal distribution data are presented with median and interquartile intervals. Mann–Whitney *U* test will be used to compare ordered categorical variables between groups.

Repeated measures ANOVA will be used to study the independent effects, main effects, and interactions of time and grouping. *p* < 0.05 is considered statistically significant. Intentionality and protocol sets will be used to analyze primary and secondary outcomes. The results of intentionality treatment analysis are compared with those of protocol set analysis to determine whether the results are consistent. Missing data are processed as multiple imputation. Adverse events will be listed and analyzed using chi-square tests or Fisher precision tests. We analyzed test results using the IBM SPSS24.0 software.

### Interim analyses {21b}

One interim analysis is to be performed when 39 patients had been enrolled. The investigators performed the analyses according to methods described in the statistical plan to ensure the robustness and validity of the analyses.

### Methods for additional analyses (e.g., subgroup analyses) {20b}

Sub-group analyses are not foreseen.

### Methods in analysis to handle protocol non-adherence and any statistical methods to handle missing data {20c}

Missing data will be addressed using multiple inference methods. Analyses are performed in the imputed datasets, and the results are summarized to ensure that the data are unbiased and the inferences are reliable.

### Plans to give access to the full protocol, participant-level data, and statistical code {31c}

The study protocol and data analysis will be obtained from the corresponding author according to the protocol.

## Oversight and monitoring

### Composition of the coordinating center and trial steering committee {5d}

The ethics committee of Zhujiang Hospital Affiliated to Southern Medical University will supervise the researchers and all levels of the study to ensure that the study follows ethical principles and protects the health of patients.

### Composition of the data monitoring committee, its role and reporting structure {21a}

A data monitoring committee is not required because of the short duration of our trial and the known low risks. But researchers will regularly analyze the data and make adjustments.

### Adverse event reporting and harms {22}

Adverse events for patients will be recorded at each follow-up visit. Adverse events are defined as all negative events of consequence that occur during the trial, regardless of their relationship to the study content, and need to be recorded when they occur. Details of the relevant event will also be recorded and a determination made as to whether it is an adverse event.

We will report and manage adverse events associated with drug injections in detail, mainly induced muscle atrophy. Participants are encouraged to keep a daily training diary to monitor their physical condition. This is a quick way to identify and mitigate any potential hazards.

### Frequency and plans for auditing trial conduct {23}

Our study will have scheduled audits every 4 weeks. The audit included data on participants’ personal information, written informed consent, and primary and secondary assessment scales. In addition, we will carefully review good practice for treatment safety, reporting of adverse events, and completeness and accuracy of data collection. Routine audits reinforced methodological rigor and ethical compliance.

### Plans for communicating important protocol amendments to relevant parties (e.g., trial participants, ethical committees) {25}

Any measures that may affect the study protocol, patient interests, or patient safety need to be modified. This includes aspects such as study objectives, study design, demographics, and sample size. All proposed changes need to be submitted to the Ethics Committee of Zhujiang Hospital Affiliated to Southern Medical University for approval. The revised protocol can proceed only after the necessary ethical approval has been obtained.

### Dissemination plans {31a}

The findings of the study will be released to participants, medical professionals, the general public, and other relevant groups through publications.

## Discussion

This trial is designed to investigate the improvement of shoulder pain after botulinum toxin or glucocorticoid injections in two groups of patients with hemiplegia. Botulinum toxin is a new method of treating neuropathic pain and has shown promising results in the treatment of neuropathic and chronic pain disorders [6, 34]. Some scholars believe that botulinum toxin can inhibit the release of inflammatory transmitters from peripheral sensory nerve endings and indirectly inhibit nociceptive sensitization of nerves [35].

Previous studies have been conducted including the study by Mahowald et al., in 2006, which evaluated the treatment of pain with intra-articular botulinum toxin [36], and the randomized controlled trial comparing intra-articular botulinum toxin injection with placebo [37]. A prospective study in Korea also compared intra-articular injection of botulinum toxin with triamcinolone acetonide for frozen shoulder [37]. Although intra-articular botulinum toxin injection is not a pioneering technique, the innovation of this study is to apply this method specifically to neuropathic pain after stroke and to examine changes in cortical activation by functional NIR spectroscopy. With the help of fNIRS to study the therapeutic mechanism, we applied it to the clinic in order to determine the degree of relief of neuropathic pain after stroke by botulinum toxin and to obtain a more objective criterion for the assessment of rehabilitation. Furthermore, we chose this controlled trial to compare the effectiveness of botulinum toxin and glucocorticoid injection therapy, which provides stronger evidence than comparisons with placebo or no treatment.

For the treatment of HSP, more published literature has injected botulinum toxin into the affected pectoralis major muscle or subscapularis to improve the angle of movement of the patient’s shoulder joint by decreasing muscle tone, thereby reducing pain triggers. It has been suggested that the effect of botulinum toxin on pain is independent of its effect on muscles and acts directly on sensory neurons [38, 39], considering that intra-articular cavity injections can obtain longer-term pain relief [40], which can effectively avoid adverse reactions such as muscle atrophy from injection into muscle tissue. This is the main reason why we chose joint cavity injections as our target.

We will primarily attempt to quantify the relief of the patient’s shoulder pain using VAS. To observe the improvement in pain in relation to the oxyhemoglobin concentration in the corresponding brain regions, we will apply the fNIRS to quantify the corresponding brain regions of the patients. fNIRS is particularly suitable for predicting changes in pain intensity. Quantifying changes in cortical activation associated with nociception by fNIRS. Related studies have also confirmed the involvement of the PFC and S1 brain regions in pain processing [41]. We hoped that changes in blood flow activity in the corresponding brain regions would provide objective data to support shoulder pain relief to complement patients’ subjective report of pain scores. The agreement between subjective perception of pain and objective HbO in subjects measured using fNIRS is more consistent. The Fugl-Meyer assessment examined whether pain reduction improves motor function, an important functional outcome in post-stroke rehabilitation.

A limitation of this study is the lack of a control group receiving sham injections of placebo. The short follow-up period of 4 weeks is another limitation. Long-term outcome data could help assess the durability of injection pain relief. In conclusion, this trial is research to assess the effectiveness and security of ultrasound-guided botulinum toxin injections in the treatment of patients with HSP. The results of this research will be important for clinicians to prescribe as a treatment option, and the scientific and methodologically rigorous design of this trial is expected to provide a reliable reference for the future application.

Future studies could explore intra-articular botulinum toxin injections in combination with rehabilitation programs (e.g., physiotherapy or occupational therapy) to optimize functional outcomes. Other neuroimaging methods, such as fMRI, may also provide further insights into the central mechanisms of hemiplegic shoulder pain.

## Trial status

At the time of submission, this study has completed ethical registration and has not yet started the trial. The protocol version number is 04, and the date is February 13, 2023. The recruitment start date will be March 1, 2024, and the estimated end date will be September 1, 2025.

## Data Availability

Data will be available from the corresponding author upon reasonable request.

## Abbreviations

HSP: Hemiplegic shoulder pain
fNIRS: Functional near-infrared imaging
VAS: Visual analog scale
HBO: Oxyhemoglobin concentration
DLPFC: Dorsolateral prefrontal area
M1: Primary motor area
S1: Primary sensory area
ROM: Passive range of motion
